# Effects of Monoterpenes of *Trachyspermum ammi* on the Viability of Spermatogonia Stem Cells In Vitro

**DOI:** 10.3390/plants9030343

**Published:** 2020-03-09

**Authors:** Sahar Omidpanah, Fereshte Aliakbari, Seyed Mohammad Nabavi, Mahdi Vazirian, Abbas Hadjiakhoondi, Mahdieh Kurepaz-mahmoodabadi, Azadeh Manayi

**Affiliations:** 1 Medicinal Plants Research Center, Faculty of Pharmacy, Tehran University of Medical Sciences, 1417614411 Tehran, Iran; sahar_omidpanah@yahoo.com (S.O.); nabavi208@gmail.com (S.M.N.); vazirian_m@tums.ac.ir (M.V.); abbhadji@tums.ac.ir (A.H.); m-mahmoodabadi@farabi.tums.ac.ir (M.K.-m.); 2 Infertility & Reproductive Health Research Center, Shahid Beheshti University of Medical Sciences, 1417614411 Tehran, Iran; Fereshtehaliakbary@yahoo.com; 3 Applied Biotechnology Research Center, Baqiyatallah University of Medical Sciences, 1417614411 Tehran, Iran; 4 Department of Pharmacognosy, Faculty of Pharmacy, Tehran University of Medical Sciences, 1417614411 Tehran, Iran

**Keywords:** apiaceae, stem cells, *T. ammi*, thymol

## Abstract

*Trachyspermum ammi* (Apiaceae) plants have several medicinal and condimentary applications and are considered an aphrodisiac agent in Iranian Traditional Medicine. Thus, the present study aims to evaluate the effects of oil from Iranian *T. ammi* plants on the viability of spermatogonial stem cells in vitro. The essential oil of *T. ammi* fruits was extracted by hydrodistillation, and the amount of thymol was calculated by a gas-chromatography method. Spermatogonial stem cells were isolated from the testes of mice using enzyme digestion. Real-time polymerase chain reaction (RT-PCR) was applied to assess the gene expressions of promyelocytic leukemia zinc finger protein (*Plzf*), DNA-binding protein inhibitor (*ID-4*), tyrosine-protein kinase (*c-Kit*), B-cell lymphoma 2 (*Bcl2*) and Bcl2-associated X protein (*BAX*). The number and diameter of colonies were also measured in the treated cells. The amount of thymol in the oil was 130.7 ± 7.6 µg/mL. Flow cytometry analysis showed that 92.8% of all cells expressed stimulated by retinoic acid 8 (*Stra8*), a spermatogonial stem cell marker. Expression of *Plzf* and ID-4 genes significantly increased in the treatment groups, while *c-Kit* and *BAX* decreased, and *Bcl2* increased in the presence of essential oil. The numbers and diameters of cells were also improved by the application of the plant oil. These data indicated that monoterpenes from the oil of *T. ammi* improved the quality and viability of spermatogonia cells in the cell culture.

## 1. Introduction

*Trachyspermum ammi* (L.) Sprague ex Turrill (Apiaceae) is an annual plant distributed in Iran, Afghanistan, Pakistan, India and northern Africa [1]. The plant has a striated stem with a white inflorescence compound called umbel, and is widely grown in arid and semi-arid areas [2].

As a powerful toolbox, nutraceuticals can be used in the prevention and treatment of diseases beyond diet and before drugs, especially in subjects who may not be eligible for treatment with conventional medications [3]. For several nutraceuticals in the plant kingdom, it is recognized that understanding their safety and mechanism of action will provide new vistas to new therapeutic agents [3,4]. The brown, seed-like fruits of *T. ammi* with their bitter and pungent taste are used as nutraceuticals for medicinal and condimentary purposes [5].

The composition of the essential oil of *T. ammi* fruit, a concentrated hydrophobic liquid containing volatile chemical compounds, was analyzed several times in previous studies using gas-chromatography (GC) or gas-chromatography-mass spectrometry (GC-MS) methods. The monoterpenes thymol, carvacrol, γ-terpinene, cymene, and limonene were identified as the main compounds of the oil of the fruit [6,7,8,9,10]. The content and composition of *T. ammi* essential oil varied in previous studies. For instance, the amount of thymol in the oil obtained from fruit ranged from 17% to 71%, indicating the effect of genotype or environmental conditions [11,12]. Salt stress caused significant reductions in the weights of the plant’s parts and its fruits, whereas the concentration of fruit oil was not affected by salt stress [12]. Aside from volatile compounds, other active primary and secondary metabolites such as carbohydrates, proteins, vitamins, minerals, tannins, carotenoids, alkaloids, steroids, saponins, and flavonoids were identified in the whole plant [13]. Water soluble constituents of the fruit have also been elucidated, including one monoterpenoid, five new monoterpenoid glucosides, two aromatic compound glucosides, and two glucides [14].

The essential oil of *T. ammi* fruit suppresses the growth of Gram-negative bacteria, Gram-positive bacteria, and fungi in vitro [6]. The fruit is applied traditionally for stomach disorders such as flatulence, indigestion, colic and diarrhea. Other pharmacological and biological activities, including antioxidation, antivirus, antifungal, nematicidal, anti-inflammatory, analgesic, hepatoprotective, antiepileptic, antifever, and wound healing effects, were attributed to the plant in recent investigations [7,8,13,15,16,17,18]. Powder from the fruit and the plant’s aqueous extract showed an antihelminthic effect in a dose-dependent manner, and the extract was rich in thymol [19]. Fruit extract showed antispasmodic and antihypertensive activity by the inhibition of K^+^-induced contraction. Additionally, the extract exhibited protective activity against both paracetamol- and CCl4-induced toxicity in mice [20].

Genetic information is transmitted to the next generation by spermatogonial stem cells, which are extensively rare, composing about 0.03% of germ cells [21]. Fertility in cancer patients may be threatened due to gonadotoxic therapies, including chemotherapy and irradiation, that can affect the spermatogenesis process.Therefore, protection of germ cells can be considered an alternative strategy to conserve fertility in these patients [22]. Previous studies revealed protective effects of extracts and natural compounds on spermatogonial cells. For instance, quercetin protected spermatogonial cells in the presence of oxidant agents [23]. Additionally, the ethanolic extract of *Moringa oleifera* reduced cyclophosphamide-induced damage on spermatogonial cells [24]. Results of previous studies also revealed that the essential oil of *T. ammi* caused morphological deformities of the sperm plasma membrane and detachment of head-to-tail coiling. This suggested that the oil is suitable to be applied as a spermicidal agent in vaginal contraceptives [25,26]. However, in Iranian Traditional Medicine the plant has a reputation of having an aphrodisiac effect, with galactagogue and diuretic properties [27]. There is no research regarding the effect of the plant on spermatogonial cells; therefore, the present study aims to evaluate the effect of the plant’s oil on the viability of spermatogonial stem cells in vitro.

## 2. Materials and Methods

### 2.1. Plant Materials

*T. ammi* fruit was purchased from local medicinal plant shops in Tehran Province, Iran (2014) and identified at the herbarium of the Faculty of Pharmacy, Tehran University of Medical Sciences. In order to obtain essential oil, the fruit (100 g) was subjected to the hydrodistillation method, using a Clevenger-type apparatus. The plant material was poured into a glass balloon with sufficient distilled water (about 500 mL) and heated until boiling for 4 h. The obtained oil was dried over anhydrous sodium sulfate (Merck, Darmstadt, Germany) and stored in a sealed, dark glass vial in a refrigerator (4 ºC) until further investigation.

### 2.2. Thymol Quantification Using GC

According to the composition previously reported in our study, thymol is the major component of the plant’s oil [11]. Therefore, the amount of thymol was analyzed quantitatively using a Dani Master GC (Dani, Italy) with an OV1 column (SE54CB, 25 m × 0.25 mm internal diameter 0.25 µm film thickness), nitrogen as a carrier gas, with a split ratio of 1:30, and a flame ionization detector (FID). Temperature programming was performed from 75 °C (42 min) to 250 °C (14 min) at 15 °C/min. Injector and detector temperatures were 250 and 260 °C, respectively. Thymol with a concentration of 12.5 to 100 µL/mL was prepared to obtain the calibration curve. The oil of the fruit was injected into the column under the same conditions, and the amount of thymol was calculated using the following equations (Equations (1) and (2)):

Y = 0.0341X−0.378(1)

R^2^ = 0.98 (2)

### 2.3. Isolation of Spermatogonial Cells

Male laboratory mice (3-6 days old) were purchased from the National Medical Research Institute (NMRI) and maintained in accordance with the guidelines issued by Tehran University of Medical Sciences. Spermatogonial stem cells were isolated in two steps of enzymatic digestion from testis tissue. Phosphate buffered saline (PBS; Sigma, Munich, Germany) was applied to wash the collected testes of mice. Testes were minced into small pieces after removing the tunica albuginea and then digested in a medium containing collagenase type IV (1 mg/mL; Sigma), hyaluronidase (0.5 mg/mL; Sigma), and DNase (10 µg/mL; Sigma) in minimum essential medium alpha (MEMα; Sigma) for 20 min. The tubules were separated with shaking at 37 °C in 5% CO_2_. The cells were centrifuged at 1500× *g* for 5 min and then washed twice with PBS [28].

The project was approved by an ethical committee (Approval ID: IR.TUMS.NIHR.REC.1398.005) that certified the project was in accordance with ethical principles and standards for conducting research.

### 2.4. Cell Enrichment Percentage

Flow cytometry was performed to assess *Stra8*-positive cells that had been isolated from testes. A total of 10^6^ cells were suspended in 100 µL of PBS/fetal bovine serum (FBS), and 10 µL of a primary antibody (anti-*Stra8* antibody, cat. no. ab49602, Abcam, Cambridge, UK) was added to the cell suspension (20 min, 4 °C). Cells were then washed with 1 mL of PBS/FBS. Subsequently, 10 µL of a secondary antibody (donkey antirabbit, cat. no. ab6798, Abcam) conjugated with fluorescein isothiocyanate (FITC) was added (20 min, 4 °C). Cells used as controls were not treated with antibodies. Cells were kept in the dark on ice until analysis by flow cytometry [29].

### 2.5. Spermatogonial Cell Culture

The cells, divided into 4 groups (3 treatment groups and 1 control group), were cultured for 2 weeks. Basic medium cultures contained MEMα, nonessential amino acids (Invitrogen, Carlsbad, CA, USA), 10% FBS, 0.1 mM 2-mercaptoethanol (Sigma), 10^3^ U/mL human recombinant leukemia inhibitory factor (LIF; B&D, Franklin Lakes, NJ, USA), 100 U/mL penicillin, and 100 µg/mL streptomycin (both from Sigma). The essential oil of *T. ammi* was added to the cell cultures in amounts of 10, 20, and 30 µL. The culture media were changed every other day and stored at 35 °C in an atmosphere humidified with 5% CO_2_. The diameter and number of colonies were evaluated using an inverted microscope (Ziess, Jena, Germany), and captured images were processed by imageJ software [29].

### 2.6. Real-Time Polymerase Chain Reaction (Real-Time PCR)

The pluripotency of the spermatogonial cells was examined by evaluating the expression levels of *ID-4* and *Plzf* alongside the apoptosis regulators *BAX*, *Bcl2*, and tyrosine-protein kinase (*c-Kit*) using real-time PCR. The sequence of primers is indicated in Table 1. Total RNA was extracted by an RNase kit (Accuzole Total RNA Extraction Kit, K-3090, Kentucky, USA) according to the manufacturer’s instructions. One microgram of total RNA was used in the synthesis of cDNA using reverse a transcription kit (Thermo Scientific, K1621, Kentucky, USA). Real-time PCR was performed with 40 reaction amplifications using 7500 fast real-time PCR and the detection of SYBR Green (Applied Bioscience, Carlsbad, CA, USA). All samples were normalized against glyceraldehyde-3-phosphate dehydrogenase (GAPDH) (internal control) using the comparative CT method (ΔΔCT) [28].

### 2.7. Viability of Spermatogonial Cells

The cell count was performed using a hemocytometer. A cell suspension (20 µL) with phosphate buffer solution (80 µL) was colored with 100 µL of trypan blue 0.4% for 5 min. Viable and dead cells (blue colored cells) of the mixture were counted in 10 µL.

### 2.8. Statistical Analysis

Data are presented as mean ± standard deviation (SD) of three independent experiments. The statistical significance between the mean values was determined by one-way analysis of variance (ANOVA) followed by a Tukey post hoc test with *p* < 0.05 as the statistically significant criterion.

## 3. Results

### 3.1. Essential Oil

Fruits of *T. ammi* (100 g) yielded 3.5 mL of yellow colored essential oil with a gravity of 0.97 mg/mL. As it was indicated in our previous study, 13 monotrepenes composed 99.1% of the oil, as identified using the gas-chromatography-mass spectrometry (GC-MS) method. Thymol (74.2%), *p*-cymene (16%), and γ-terpinene (7.1%) were the main constituents of the oil [11]. Therefore, the amount of thymol as a major component was assayed in the oil using the GC method. According to the thymol calibration curve, the amount of thymol was 130.7 ± 7.6 µg/mL in the essential oil of *T. ammi* fruit.

### 3.2. Spermatogonial Cell Culture

Flow cytometry analysis showed that 92.8% of the isolated cells from mice testes expressed *Stra8*, a spermatogonial stem cell marker. The diameters of all the colonies treated with *T. ammi* oil were significantly higher than those of the control group (Table 2), while there was no significant difference observed between the treatment groups in the increase of colonies’ diameters. The respective diameters of the control group were 48.6 ± 0.64 and 195.57 ± 1.36 mm in the first and second weeks. In the first week, colony diameters in the treatments of 10, 20, and 30 µL of the oil were 78.42 ± 2.31, 89.26 ± 0.48, and 79.71 ± 0.68 mm, respectively. The diameters of the colonies increased similarly, and 20 µL of the oil induced colony diameters (283.53 ± 8.05 mm) that were higher than the other treatments in the second week of the study. Colony diameters in the presence of the oil (10 and 30 µL) were increased to 272.26 ± 11.23 and 276.35 ± 0.48 mm, respectively. The number of colonies was significantly increased in the presence of all concentrations of the plant oil in both weeks of observation compared with control group (*p <* 0.05). Similar to the diameters of the colonies, the number of colonies increased in the presence of 20 µL of oil, from 17 ± 1.1 in the first week to 28 ± 1.2 in the second week, compared to the other groups. In the other treatment groups, i.e., 10 and 30 µL of oil, the colony numbers enhanced after two weeks to 25 ± 1.7 and 26 ± 1.1, respectively.

### 3.3. Gene Expression

**Figure 1:**
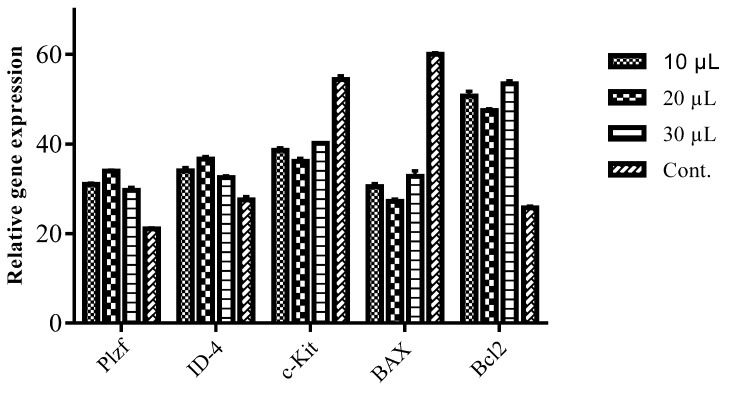
Expression patterns of *Plzf*, *ID-4*, *c-Kit*, *BAX*, and *Bcl2* genes after treatment with different amounts of *T. ammi* fruit oil (10, 20, and 30 µL). Expressions of *Plzf* and *ID-4* were increased after treatment with oil, while the expression of *c-Kit* was decreased. Expression levels of *BAX* and *Bcl2* were decreased and increased after treatment, respectively. Stars show significant differences between treatment groups and the control group (*p* < 0.05).

A significant increase in the expression of genes related to the undifferentiated state, *ID-4* and *Plzf*, in spermatogonial cells in the treatment groups of 10, 20, and 30 µL of oil was observed, which was more than that in control group (*p* < 0.05). The enhancement of gene expression was higher in the treatment with 20 µL of oil in comparison to the others (Figure 1). Meanwhile, the expression of the *c-Kit* gene (differentiated gene) in all the treatment groups was significantly decreased compared to the control (*p <* 0.05), and greater amount of decrease observed in the presence of 20 µL of the oil than in the two other treatment groups. There was a significant decrease in the expression level of the *BAX* gene (pro-apoptotic) in the presence of *T. ammi* oil in comparison with the control group (*p* < 0.05). The highest decrease in the level of the *BAX* gene was obtained with 20 µL of the fruit oil, but treatments with 10 and 30 µL of the oil also considerably lowered the expression of the *BAX* gene. After the oil treatment, expression of *Bcl2* increased significantly in the presence of the oil compared with the control group (*p <* 0.05). All the oil amounts raised the expression of *Bcl2*, and the oil at an amount of 30 µL affected the expression of *Bcl2* (antiapoptotic) more than the other treatments.

## 4. Discussion

Several beneficial effects have been attributed to *T. ammi*, such as aphrodisiac properties, which led us to evaluate the effect of *T. ammi* oil on the viability of spermatogonial cells in vitro. The results of the present study revealed that like previous reports, thymol composed the main part of the fruit oil of *T. ammi*. Different amounts of thymol were presented (17% to 71%) in previous analyses [11], while the oil tested on spermatogonial cells in the present study contained 74.2% thymol. Thus, thymol as a major component was assayed in the oil using the GC method according to the thymol calibration curve.

*Stra8* was expressed in 92.8% of the cells isolated from the testes of mice, which is acceptable according to a previous study [29]. All the oil treatments (most of all, 20 µL of the oil) increased the diameter and number of colonies significantly more than the control group. Antioxidants can suppress the deteriorative effects of reactive oxygen species (ROS) and oxidative stress that can affect cell membranes, structural proteins, enzymes, and nucleic acids [30]. As it was indicated, thymol composed the main part of the tested oil, which displayed favorable effects on spermatogonial cells in the medium. Thymol exhibits antioxidation activity by inhibiting free radicals, chelating metal ions, and enhancing endogenous antioxidants, dependent or independent of enzymes [31]. In addition, γ-terpinene, which is a non-phenolic monoterpene, has antioxidant properties [32]. The results of a previous study indicated that cryopreservation and antioxidants such as α-tocopherol (belonging to the vitamin E family) and catalase (an enzyme in peroxisomes that converts H_2_O_2_ to H_2_O and O) increased the number and diameter of colonies. The obtained results are attributed to the presence of the mentioned antioxidants in the cell culture [22].

Gene expressions were analyzed after 2 weeks of the treatment with *T. ammi* oil, indicating that the oil influenced gene expressions in spermatogonial cells. The expression of *BAX* and *Bcl2* genes was changed in favor of reduction of apoptosis in the cells. Markers of spermatogonial cells, *ID-4* and *Plzf*, were overexpressed in the presence of the oil, whereas expression of the *c-Kit* gene was reduced. According to the data of another study, treatment of Chang liver cells with thymol suppressed apoptosis induced by oxidative stress, through the reduction of expression of the *BAX* gene and induction of the *Bcl2* gene. It was concluded that thymol protected cells via the inhibition of ROS, increases in glutathione, and decreases in malonyl aldehyde [33]. Thymol’s antioxidation and free radical-scavenging abilities were associated with the protective capacity of the compound against damage induced by UV radiation on human keratinocyte cell lines (HaCaT) [34]. The compound at quantities up to 250 µM did not show mutagenic or genotoxic effects using Ames and Comet assays [35]. However, treatment of glioblastoma cells with thymol caused apoptosis via the release of Ca^2+^ from the endoplasmic reticulum [36]. Results of another study described that the concentration of thymol and carvacrol (isomer of thymol) is crucial in their activity as pro-oxidants or antioxidants in Caco-2 cells [37]. Both thymol and carvacrol at lower concentrations showed antioxidant activity. It is common for phenolic compounds to show antioxidant and pro-oxidant properties depending on their concentrations [37]. In our study, treatment of spermatogonial cells with 20 µL of the oil provided better results, which implies the importance of concentration in the preservation of spermatogonial cells using *T. ammi* oil.

## 5. Conclusions

The antioxidant abilities of monoterpenes such as thymol and γ-terpinene, along with other minor components in essential oil of the fruit of *T. ammi*, may be responsible for preserving and improving the viability of spermatogonial cells of mice through induction of *Bcl2* and inhibition of *BAX*. Therefore, *T. ammi* oil can be a promising agent in the conservation of fertility in patients who suffer from malignancies and/or the fruits can be used as an appropriate nutraceutical formulation for the aforementioned purpose. Our results merely support the beneficial effect of *T. ammi* oil in cell culture. Further in vivo and trial studies are recommended concerning the protective properties of the plant.

## Figures and Tables

**Table 1:** Primers used in analyses of the effects of *T. ammi* oil on spermatogonial cells.

Gene Name	Sequence	Porduct Size	Annealing Temperature
Bcl2	F, 5’-GGGGTCATGTGTGTGGAG-3’ R, 5’-TCACTTGTGGCCCAGGTA-3’	261	57.28
Bax	F, 5’- CTGGATCCAAGACCAGGGTG-3’ R, 5’-CCTTTCCCCTTCCCCCATTC-3’	251	58.14
ID-4	F, 5’-TCCCGCCCAACAAGAAAGTC-3’ R, 5’-TCAGCAAAGCAGGGTGAGTC-3’	102	60.54
Plzf	F, 5’-CGTTGGGGGTCAGCTAGAAAG-3’ R, 5’-CACCATGATGACCACATCGC-3’	301	57.14
c-Kit	F, 5’-AACAACAAAGAGCAAATCCAGG-3’ R, 5’-GGAAGTTGCGTCGGGTCTAT-3’	200	57.67
GAPDH	F, 5’-AGCAAGGACACTGAGCAAGAG-3’ R, 5’-TCGTTCCTCTGATCGTTTCC-3’	151	60.53

*Bcl2:* B-cell lymphoma 2, *BAX*: Bcl2-associated X protein, *ID-4*: DNA-binding protein inhibitor, *Plzf*: promyelocytic leukemia zinc finger protein, *c-Kit*: tyrosine-protein kinase, GAPDH: glyceraldehyde-3-phosphate dehydrogenase.

**Table 2:** Diameters and numbers of colonies of spermatogonial cells treated with three different concentrations of *T. ammi* oil after 2 weeks. Results are presented as mean ± SD.

Treatment	Numbers of Colony		Diameters of Colony (mm)	
	1^st^ Week	2^nd^ Week	1^st^ Week	2^nd^ Week
10 µL	13 ± 1.5	25 ± 1.7	78.42 ± 2.31	272.26 ± 11.23
20 µL	17 ± 1.1	28 ± 1.2	89.26 ± 0.48	283.53 ± 8.05
30 µL	13 ± 1.1	26 ± 1.1	79.71 ± 0.68	276.35 ± 0.48
Cont.	7 ± 1.5	15 ± 1.2	48.60 ± 0.64	195.57 ± 1.36
